# Lytic Patella

**DOI:** 10.1097/JS9.0000000000003002

**Published:** 2025-07-08

**Authors:** De-an Qin, Jie Yuan, Yan-Yan Dong

**Affiliations:** a Department of Orthopedics Shanxi Provincial People’s Hospital, Taiyuan, China; b Department of Medical Imaging Center Shanxi Provincial People’s Hospital, Taiyuan, China; c Department of Pathology Shanxi Provincial People’s Hospital, Taiyuan, China

**Keywords:** diagnosis, imaging, patella, tumor

## Abstract

Lytic patellar lesions may occur in a variety of conditions, from commonly seen developmental, degenerative, traumatic, metabolic, infectious, iatrogenic to uncommon benign and malignant neoplasms. Patellar metastasis is rare. Due to the low incidence and the non-specific symptoms of metastatic anterior knee pain, delayed diagnosis is common and the prognosis is poor. It is worthwhile to remind clinicians and radiologists the possibility of patellar metastasis in patients with a history of previous malignancy presenting with an intractable anterior knee pain. We proposed the diagnosis and differential diagnosis for patellar metastasis. The diagnosis may be challenging in patellar giant cell tumor, chondroblastoma, aneurysmal bone cyst, metastasis, and gout. MRI is the most sensitive in detecting bone metastasis, delineating intraarticular invasion and planning surgical boundary. Arthrocentesis fluid cytological examination is useful as a rapid and simple diagnostic method. Multimodal imaging analysis may help make a definite diagnosis.

Lytic patellar lesions may occur in a variety of conditions, from commonly seen developmental, degenerative, traumatic, metabolic, infectious, iatrogenic to uncommon benign and malignant neoplasms^[1]^. As the largest sesamoid bone in the body, patellar neoplasms are uncommon due to the relatively poor blood supply to the patella comparing with spine, pelvis, and long bones. Giant cell tumor (GCT), chondroblastoma (CB) and aneurysmal bone cyst (ABC) are three most common benign types, together accounting for almost 90% of patellar neoplasms. Osteosarcoma, chondrosarcoma, and Ewing’s sarcoma are three most common primary malignant types. Metastatic patellar tumors are rare and the most common site of primary cancer is the lung, followed by breast, kidney, and esophagus^[2]^. Due to the low incidence and the insidious non-specific symptoms of oncologic anterior knee pain, delayed diagnosis or misdiagnosis is common and the prognosis is poor. It is worthwhile to remind clinicians and radiologists the possibility of patellar metastasis in patients with a history of previous malignancy presenting with an intractable anterior knee pain. During the preparation of this work, the authors did not use the AI and AI-assisted technologies^[3]^.

Age is one of the factors for differential diagnosis on the lytic patellar lesions. GCT occurs in the adults. CB occurs in the epiphyses or apophysis of skeletally immature adolescents. ABC predominantly affects children and young adults. Patellar metastasis usually occurs in patients older than 40 years. Gout affects up to 10 times more males than females and female gout typically occurs in post-menopause elderly women with chronic renal dysfunction and hyperuricemia taking diuretics.

**HIGHLIGHTS**

- Knee is the most frequently involved among all the joint metastasis. It is worthwhile to remind clinicians and radiologists the possibility of patellar metastasis in patients with a history of previous malignancy presenting with an intractable anterior knee pain. Multimodal imaging analysis may help make a definite diagnosis.

Imaging features of CT and MRI may help narrow the differential diagnosis including lesion margin, bone expansion, cortical destruction, matrix mineralization, and soft tissue invasion^[4]^ (Fig.1a, b). GCT may appear patellar expansion with cortical thinning and intralesional soap-bubble-like changes. CB appears well-defined geographic lytic lesion with scattered intralesional calcifications. ABC typically appears a multi-chamber lytic expansile lesion with fluid levels. ABC secondary to CB or GCT is diagnosis-challenging due to the overlapping imaging features. The punched-out bone erosions and intraarticular effusion with quadriceps or patellar tendon thickening in patellar gout may mimic patellar metastasis^[5]^. MRI is the most sensitive in detecting bone metastasis, delineating soft tissue invasion and planning surgical boundary. It can show the intraarticular tumor invasion with three direct signs (intrasynovial tumor tissue, destruction of cartilage/bone, invasion of capsular/ligamentous insertions), and three indirect signs (signal alterations, synovial enhancement, joint effusion)^[6]^.

**Figure 1.**
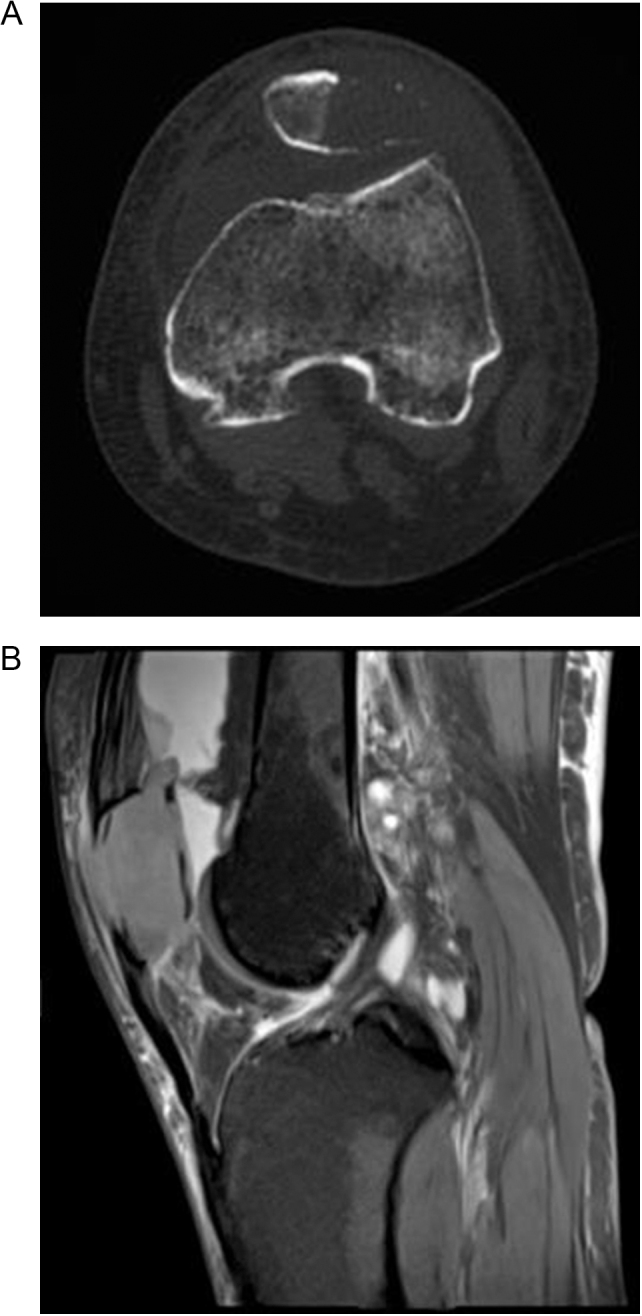
(A) Axial knee CT showed an eccentrically-based lytic patellar lesion with cortical and cartilaginous erosion. (B) Sagittal proton density-weighted MRI showed a worm-eaten patellar lesion with homogenous intermediate signal intensity and without internal calcification, septum or fluid level, combining with suprapatellar pouch effusion, quadriceps tendon insertion thickening and popliteal lymph nodes swelling. Arthrocentesis fluid cytological microscopic examination showed some sparse tumor cells and cervix-derived patellar metastasis was diagnosed.

Knee is the most frequently involved among all the joint metastasis (>50%)^[7]^. Malignant joint effusion is a sensitive indicator of intraarticular tumor invasion, with a sensitivity of 91.3% but specificity of only 35%^[7]^. Arthrocentesis fluid may appear bloody in one-half of malignant cases and cytological examination is positive in only one-half of cases. So, the absence of tumor cell in arthrocentesis fluid does not rule out an intraarticular tumor invasion. Analysis of the arthrocentesis fluid has also been utilized as a rapid, simple, and routine diagnostic method. Imaging-guided patellar core needle biopsy is considered a reasonable alternative if synovial fluid cytodiagnosis is uncertain or negative. Arthrocentesis aspiration and needle biopsy should first be attempted prior to incisional biopsy for diagnosis.

The management of patellar metastasis involves a multidisciplinary and individualized approach^[8]^. Surgery is the preferred treatment for patellar metastasis. The burden of metastatic involvement and survival expectancy define the surgical extent. For the solitary patellar metastasis, patellectomy and extensor mechanism reconstruction with negative margins may improve the prognosis. For the extensive intraarticular tumor invasion, an above-knee amputation or less invasive palliative surgery such as curettage and cement packing might be sufficient for pain control.

## Data Availability

All data, models, or code generated or used during the study are available from the corresponding author by request.
